# Positive Effects of Conscious Connected Breathing Exercises on Sexual Function, Sleep, and Mood in Women with Fibromyalgia Syndrome Compared to Controls

**DOI:** 10.31138/mjr.080925.ers

**Published:** 2026-04-21

**Authors:** Gulsum Uysal, Gulşah Yaşa Ozturk, Burhan Fatih Kocyiğit, Halenur Colak

**Affiliations:** 1 Department of Obstetrics and Gynaecology, University of Health and Science Adana City Training and Research Hospital, Adana, Turkey;; 2 Department of Physical Medicine and Rehabilitation, University of Health and Science Adana City Training and Research Hospital, Adana, Turkey

**Keywords:** breathing exercises, fibromyalgia, sexual dysfunction, stress, psychological, sleep initiation and maintenance disorders

## Abstract

**Aim:**

Fibromyalgia syndrome (FMS) is a chronic rheumatic disorder characterised by body pain, decreased pain threshold and psychological distress. Our aim is to investigate the effects of breathing exercises on sexual function, sleep, and mood in FMS.

**Methods:**

This is a cross-sectional study comparing psychological assessments conducted prior to and following breathing exercises. Participants completed the Fibromyalgia Impact Questionnaire (FIQ), Hospital Anxiety and Depression Scale (HADS), and Jenkins Sleep Rating Scale (JSS) and Female Sexual Function Index (FSFI). Scores were analysed for comparison to controls.

**Results:**

A total of 56 women were enrolled to the study (30 intervention group, 26 controls). The mean age was 50.79 ± 9.06 years in intervention group. There were no statistical difference between groups occupational status (p > 0.05). The FIQ, HADS-depression, HADS anxiety, JSS and FSFI scores were similar between groups before breathing exercises (p > 0.05). FIQ, HADS depression- HADS anxiety, JSS scores after breathing exercises were better comparing to controls, p<0.001, p<0.001, p=0.002 and p=0.001, respectively. Although all symptoms were alleviated, the FSFI scores were not higher than FSD (Female sexual dysfunction) cut off value in intervention group after breathing exercises (p=0.033). The total FSFI score less than 26.55 was the cut-off for FSD. All of the participants had FSD.

**Conclusion:**

Breathing exercises can positively influence quality of sleep, anxiety, depression, and sexual life in patients with FMS. Incorporating breathing exercises into the curriculum may be considered to balance the quality of life for women with FMS.

## INTRODUCTION

Fibromyalgia syndrome (FMS) is a chronic rheumatic disorder characterised by widespread body pain, sensitivity in certain anatomical regions, decreased pain threshold and psychological distress.^1,2^ FMS is accompanied by sleep disturbances, fatigue, and cognitive disorders, as well as somatic and psychological signs.^2^ Women experience the condition up to six times more frequently than men.^3^ The most frequently affected population is middle-aged (40–60 years old) female patients.^4^ Treatment approach include pharmacological and non-pharmacological methods, and they are recommended in combination or separately.^5,6,7^

Dysmenorrhoea, chronic pelvic pain, endometriosis, and dyspareunia can accompany FMS and trigger sexual dysfunction.^8^ In FMS, which is a disorder related to stress, the hypothalamic-pituitary-adrenal (HPA) axis is proven to play a role in etiopathogenesis, and impaired balance of autonomic nervous system (ANS), higher cortisol levels, particularly in the morning, have been linked to changes in the body’s natural daily rhythm.^9^ Low serotonin levels are also effective in these hormonal changes.

Studies have demonstrated the reduction of cortisol and stress hormones through breathing exercises.^10^ Our aim in this study is to investigate the effects of conscious connected breathing (CCB) exercises on sexual function, sleep, and mood in fibromyalgia patients. By experiencing and learning breathing exercises, people can do it on their own, wherever they want, without the need for a specific tool or examination, and this could change their lifestyle.

## METHODS

This is a cross-sectional study, that comparing psychological assessments conducted prior to and following breathing exercises, administered to female patients with FMS between December 2024 and April 2025. Volunteer female patients, aged between 18 and 65 diagnosed with FMS,^11^ who applied to the Adana City Training and Research Hospital Physical Medicine and Rehabilitation outpatient clinic were included in our study. All patients were in the stable period of the disease. Individuals diagnosed with schizoaffective illness, those utilising antipsychotic medications, and pregnant participants were eliminated. Participants who did not finish three times exercises were also excluded.

Natural CCB exercises were conducted 3 times monthly (every Monday) for approximately 1.5 hours from 16:30 to 18:00 pm by qualified breathing coach (GU). Volunteer participants were recruited randomly. Initially, they were apprised of the study’s objectives and methodologies. Secondly, a physical therapy and rehabilitation expert (GYO) gathered sociodemographic data to ascertain admission criteria. Demographic information, including the duration of follow-up since the diagnosis of FMS, chronic disease history, medication history, age, body mass index, and marital status, were solicited and documented. Participants completed the Fibromyalgia Impact Questionnaire (FIQ), Hospital Anxiety and Depression Scale (HADS), and Jenkins Sleep Rating Scale (JSS) and Female Sexual Function Index (FSFI) before and after the CCB exercises. The results of the pre- and post-surveys were compared. The control group consists of women diagnosed with FMS who did not participate in breathing exercises. The assessments were administered to the participants who completed following three exercises.

A signed written informed consents were obtained, and the self-administered assessment and inquiries were recorded from all subjects. Approval to conduct the study was received from the appropriate human participants’ ethics committee (Ethics Committee of University of Health and Science Adana City Training and Research Hospital, Approval Number: 253; Date: 05.12.2024) and it was registered with ClinicalTrials. gov under identification number NCT07030374. The research was performed in accordance with the principles of the Declaration of Helsinki, as revised in 2013.

## ASSESSMENT SCALES

### The Fibromyalgia Impact Questionnaire (FIQ)

This is a measurement instrument first developed by Burckhardt et al.^12^ Sarmer et al.^13^ conducted the Turkish validity and reliability evaluation. The purpose of the FIQ is to assess the effects of fibromyalgia. The evaluation encompasses information regarding the patient’s physical capabilities, weariness, energy levels, pain, sleep disruptions, and psychological condition. The questionnaire comprises ten principal inquiries. The maximum attainable score is 100, signifying a more severe clinical condition as values rise.

### Hospital Anxiety and Depression Scale (HADS)

It was first developed by Zigmond and Snaith in 1983 and subsequently modified for Turkish application by Aydemir et al.^14,15^ It is frequently utilised in medical settings, especially in hospitals and clinics, to assess the psychological state of persons. The HADS comprises two subscales that assess anxiety and depression. The HADS typically comprises 14 items, evenly divided into seven questions for the anxiety subscale and seven for the depression subscale. The total scores between 0–7, 8–10 and above 11, were normal, borderline and abnormal; respectively. Each inquiry relates to a certain emotional state, enabling patients to evaluate the emotional experiences they have encountered during a designated period, typically within the past week.

### Jenkins Sleep Rating Scale (JSS)

Jenkins et al.^16^ developed the scale, a tool for assessing sleep disorders. Duruöz et al.^17^ performed a validity and reliability research in Turkey. The questionnaire evaluated concerns like “difficulty initiating sleep, frequent nocturnal awakenings, inability to maintain sleep without interruptions, and persistent fatigue upon awakening after a typical sleep duration.” Higher JSS scores indicate lower sleep quality.

### Female Sexual Function Index (FSFI)

The FSFI is a valid and reliable structured questionnaire including six domains (desire, subjective arousal, lubrication, orgasm, satisfaction, pain), comprising 19 items that assess female sexual function.^18^ The Turkish adaptation of the FSFI was established as valid and dependable for Turkish women by Oksuz et al.^19^ FSFI higher score means better sexual function (maximum score 36).

### Breathing exercises

CCB is breathing through the mouth without pausing between inhalation and exhalation, accompanied by music. It could be practiced in a group or individually. Group breathing exercises commenced following the participants’ education on breathing and the completion of pre-session assessments. Exercises started with a jumping dance to stimulate respiration prior to the breathing. During rhythmic jumping accompanied by music, referred to as “kundalini breathing,” the arms are elevated during inhalation and down upon exhalation and respiration occurs through the mouth.

During group breathing exercises, each participant selects a mat and reclines supine with knees flexed and eyes closed. Pillows and blankets were additionally arranged. The essential aspects of these breathing exercises are: inhale and exhale in a continuous way without interruption, and breathe as deeply as possible. During the breathing session, respiration occurs through the mouth to facilitate increased airflow into the body. Breathing exercises endure for roughly 45 minutes accompanied by music. Breathing coaches motivate participants and remind them to “maintain respiration, regardless of circumstances.” They employed ways to facilitate participants in maintaining a natural rhythmic breathing pattern, similar to that of a baby. Groups consist of 6 to 10 individuals. The practice concludes with a 10-minute meditation in the same posture, synchronised with each individual’s nasal respiration. Mouth breathing should be avoided outside of exercises.

### Statistical Analysis

All statistical analyses were conducted using SPSS software version 23.0 (IBM Corp., Armonk, NY, USA). The Shapiro-Wilk test was employed to evaluate the normality of continuous variables. Continuous variables exhibiting a normal distribution were reported as mean ± standard deviation (SD), whereas those with a non-normal distribution were presented as median (minimum–maximum). Categorical variables were summarised using numbers (n) and percentages (%). To facilitate comparisons between the intervention and control groups, the Independent Samples t-test was employed for continuous variables that demonstrated a normal distribution, whereas the Chi-square test was utilised for categorical variables. The Mann-Whitney U test was applied for continuous variables that do not conform to a normal distribution (such as the number of pregnancies and children).

Within-group comparisons of pre- and post-intervention data in the intervention group were conducted using the Paired Samples t-test for continuous variables.

A p-value of <0.05 was considered statistically significant.

## RESULTS

A total of 56 participants were enrolled to the study (30 intervention group and 26 controls). All of them were women. The mean age of participants was 50.79 ± 9.06 years in intervention group. There was no statistical difference between groups in terms of age, educational, occupational, and smoking status (p > 0.05). Most of the participants’ educational status was university and more than half of them were working. **Table 1** presented the demographic and occupational characteristics.

**Table 1.** The baseline demographic and occupational features of the groups.

	**Intervention group n=30**	**Control group n=26**	**p**

Age*	50.79 ± 9.06	49.40 ± 10.12	0.164

**Educational status (n) (%)**			
**Primary School**	1 (3.3)	3 (11.5)	
**Middle School**	1 (3.3)	0 (0)	
**High School**	8 (26.7)	10 (38.5)	0.329
**University or above**	20 (66.7)	13 (50)	

**Occupational status (n) (%)**			
**Working**	21 (70)	15 (57.7)	
**Not working/Housewife**	9 (30)	10 (38.5)	0.411
**Retired**	0 (0)	1 (3.8)	

**Smoking status (n) (%)**			
**Yes**	6 (20)	7 (26.9)	
**No**	24 (80)	19 (73.1)	0.752

**Number of pregnancies^a^**	2 (0 - 4)	2.5 (0 - 8)	0.092

**Number of children^a^**	2 (0- 3)	2 (0 - 7)	0.079

*: Data are expressed as mean ± standard deviation.

a: Data are expressed as median (minimum–maximum).

n: Number; %: Percentage.

Comparison of groups in terms of clinical parameters before CCB exercises were shown in **Table 2**. The FIQ, HADS-depression, HADS anxiety, JSS and FSFI scores were similar between groups before breathing exercises (p > 0.05).

**Table 2.** Comparison of groups in terms of clinical parameters before conscious connected breathing exercises.

	**Intervention group n=30**	**Control group n=26**	**p**
**FIQ**	51.81 ± 13.43	59.08 ± 17.04	**0.080**
**HADS-depression**	7.33 ± 2.42	8.80 ± 3.66	**0.078**
**HADS-anxiety**	11.36 ± 3.79	11.07 ± 4.56	**0.796**
**JSS**	15.80 ± 19.74	12.34 ± 6.78	**0.400**
**FSFI**	19.10 ± 7.75	16.59 ± 7.62	**0.229**

FIQ: Fibromyalgia Impact Questionnaire; HADS: Hospital Anxiety and Depression Scale; JSS: Jenkins Sleep Scale; FSFI: Female Sexual Function Index.

**Figure 1.**
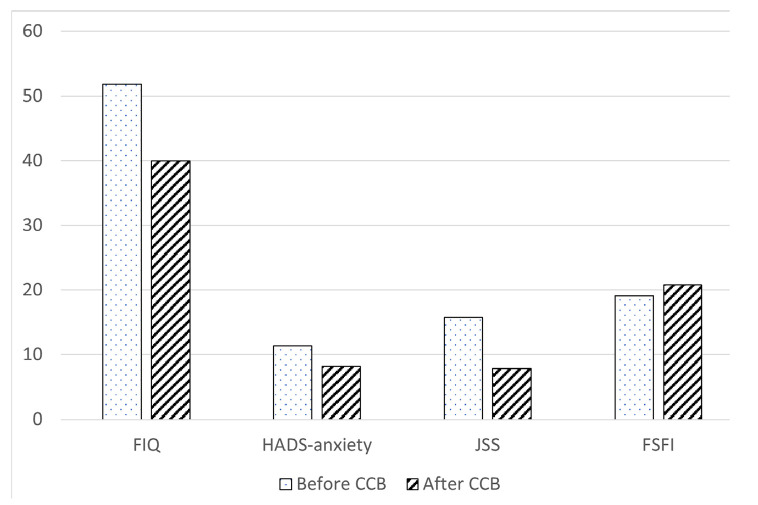
The comparision of scores before and after breathing exercises in intervention group. FIQ: Fibromyalgia Impact Questionnaire; HADS: Hospital Anxiety and Depression Scale; JSS: Jenkins Sleep Scale; FSFI: Female Sexual Function Index; CCB: Conscious Connected Breathing.

**Table 3** and **Figure 1** showed comparison of pre- and post-intervention scores in intervention group. All of the scores in assessment scales were statistically significantly changed after breathing exercises. The FIQ, HADS anxiety- HADS depression, JSS and scores were decreased significantly after the breathing session (p<0.001). HADS scores normalised, with anxiety being more pronounced. The slightly increased FSFI score was also statistically significantly different after breathing intervention (p<0.001).

**Table 3.** Comparison of pre- and post-intervention scores.

	**Pre-intervention**	**Post-intervention**	**p**
**FIQ**	51.81 ± 13.43	39.94 ± 13.13	<0.001
**HADS-depression**	7.33 ± 2.42	5.46 ± 2.25	<0.001
**HADS-anxiety**	11.36 ± 3.79	8.20 ± 2.95	<0.001
**JSS**	15.80 ± 19.74	7.90 ± 3.37	<0.001
**FSFI**	19.10 ± 7.75	20.76 ± 7.86	<0.001

FIQ: Fibromyalgia Impact Questionnaire; HADS: Hospital Anxiety and Depression Scale; JSS: Jenkins Sleep Scale; FSFI: Female Sexual Function Index.

**Figure 2.**
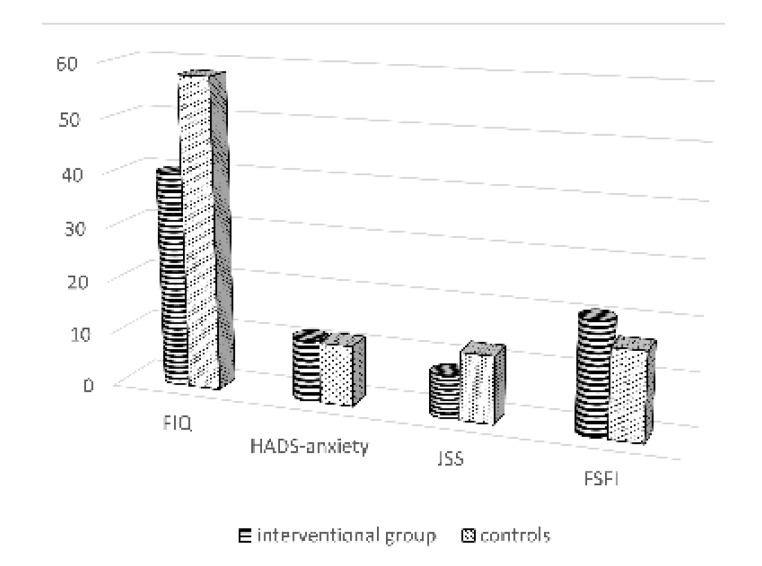
The comparision of scores between groups after the intervention. FIQ: Fibromyalgia Impact Questionnaire; HADS: Hospital Anxiety and Depression Scale; JSS: Jenkins Sleep Scale; FSFI: Female Sexual Function Index.

The comparison of scores in assessment scales after the CCB exercises between groups were shown in **Table 4** and **Figure 2**. FIQ, HADS depression- HADS anxiety, JSS scores after breathing exercises decreased statistically significantly comparing to controls, p<0.001, p<0.001, p=0.002 and p=0.001, respectively. Although all symptoms were alleviated, the FSFI scores were not higher than the FSD (Female sexual dysfunction) cut off value in intervention group after breathing exercises (p=0.033). The total FSFI score less than 26.55 was the cut-off for FSD. All of the participants had FSD.

**Table 4.** Comparison of groups after the conscious connected breathing exercises.

	**Intervention group n=30**	**Control group n=26**	**p**
**FIQ**	39.94 ± 13.13	57.99 ± 16.56	<0.001
**HADS-depression**	5.46 ± 2.25	8.96 ± 3.62	<0.001
**HADS-anxiety**	8.20 ± 2.95	11.23 ± 4.10	0.002
**JSS**	7.90 ± 3.37	12.50 ± 6.30	0.001
**FSFI**	20.76 ± 7.86	16.44 ± 6.75	0.033

FIQ: Fibromyalgia Impact Questionnaire; HADS: Hospital Anxiety and Depression Scale; JSS: Jenkins Sleep Scale; FSFI: Female Sexual Function Index.

## DISCUSSION

This study showed that CCB exercises had a statistically significantly positively affect patients with FMS including sleep quality, anxiety, depression and sexual life. Another notable result was that all participants had FSD.

Jin Ho Kang et al. quantified autonomic dysfunction in fibromyalgia patients compared to healthy controls using heart rate variability (HRV) and showed higher standard deviation of normal to normal R-R intervals in patients with FMS.^9^ It has already known that fibromyalgia shows sympathetic hyperactivity and parasympathetic hypoactivity.^20^ Oscillation in cardiac cycles is controlled by sympatho-vagal balance of the autonomic nervous system (ANS) and autonomic dysfunction is a possible mechanism linked to pain and psychosocial stress in fibromyalgia patients. The dysfunction encompasses sympathetic hyperactivity and reduced parasympathetic activity.^9^ This also results in less cardiac vagal withdrawal to gravitational stress, hence reducing orthostatic tolerance. Autonomic dys-function and unbalanced pain modulating responses are thought to be major theory of the pathophysiology of fibromyalgia. Moreover, in FMS, endorphins, which have pain-relieving properties, are also released less during exercise and exaggerated pain in fibromyalgia may be answered with muscular ischemia.^21^

Natural breathing, referred to as “Conscious Connected Breathing (CCB),” produces three fundamental effects: Physiological, psychological and spiritual effects. The physical consequences include heightened energy levels and a healthy immune system resulting from enhanced cellular oxygenation.^22^ It exerts a massaging influence on digestion, breathing, and hormonal secretion through the movement of the diaphragm muscle.^10,22,23^ The emotional and mental consequences include enhanced creativity, clarity of emotional thinking, and improved self-expression of sentiments.^10,22,23^ Deep abdominal breathing was demonstrated to activate the parasympathetic nervous system.^24^ Vagal stimulation would alter parasympathetic predominance. The heart rate elevates during breathing and diminishes after expiration. Sympathetic and parasym-pathetic stimulation, along with respiratory rate and volume, influence respiratory sinus and blood pressure oscillations.^25^ The impact of breathing exercises on stress hormones was examined; findings indicated a reduction in cortisol and an elevation in oxytocin.^10^ All mechanism of breathing impact on ANS and vagal stimulation could explain our study results for FMS.

In a recent study, they investigated breathing function in people with FMS by examining acid–base balance and compared it with healthy controls.^26^ Women with fibromyalgia exhibited significantly reduced carbon dioxide pressure (p = 0.013) and elevated lactate levels (p = 0.038) in comparison to healthy controls at the group level.^26^ Dysfunctional breathing may cause unbalanced acid-base cycle and it can be detected with the analysis of blood gases. Previous studies have suggested that altered blood gases may indicate respiratory dysfunction in chronic pain conditions such as neck and low back pain.^26,27^

Slow-paced breathing, a technique involving deliberate, measured breaths in reaction to anxiety-inducing stimuli, has demonstrated the capacity to modify autonomic nervous system (ANS) activity in several manners. They found that during normal respiration, FM patients exhibited elevated sympathetic nervous system activity and reduced parasympathetic nervous system activity in comparison to healthy, pain-free controls.^28^ They also emphasised that controlled breathing practices can modify the detrimental alterations in ANS activity that intensify indications of chronic pain. Therefore, the implementation of controlled breathing techniques could be a component of a multidisciplinary treatment strategy in FMS.

In another study, 35 women with fibromyalgia (ages 34–67 years) were randomly allocated 2 groups; breathing exercise group (n = 18), (30 minutes per session, seven times per week, for 12 weeks) and a control group (n = 17). They assessed pain threshold tolerance at tender spots using a digital pressure algometer, and evaluated FIQ scale for daily life.^29^ Their study showed that breathing exercises program (mainly focusing diaphragmatic breathing) effectively enhanced the cumulative pain threshold tolerance at tender areas especially in the upper body including the second rib, occiput, and supraspinatus. FIQ scores were also decreased after breathing exercises similar to our study results. The same study team also compared supervised and non-supervised breathing regimes on fibromyalgia and declared that supervised regimes were more helpful on pain in FMS.^30^ Moreover, in a recent study, they offered meditative diaphragmatic breathing and vagus nerve stimulation for complimentary methods in non-invasive fibromyalgia pain treatment.^31^

Female Sexual dysfunction (FSD) is a common neglected issue that negatively impacts women’s quality of life and psychological health. Living conditions and individual geographical factors vary, but the post-menopausal period is the most common time to see it.^32^ Women with FMS had lower sexual desire than healthy women.^33^ The factors for this result were thought to be age, time since diagnosis, and depressive symptoms.^32,33^ In a recent meta-analysis, 27 studies 10 of which were case-control and 17 were cross-sectional, women with FMS had distinctly worse sexual function compared to healthy women.^34^ Therefore, psychological therapies were suggested.^34^ Sexual health that involves emotional and physical health is very important component for the comprehensive treatment of FMS.

Low back pain in fibromyalgia patients has been shown to vary seasonally, with the pain decreasing in the summer. While this finding is suggested by the increased release of serotonin due to increased sunlight, it’s inevitable that the underlying cause of the pain, stress, and mood swings is a depressive tendency.^35,36^

The pain management in fibromyalgia, includes both pharmacological and non-pharmacological options. Different alternative treatment options such as exercise and physical modalities were used in fibromyalgia; the effectiveness and safety of the methods were proved.^37^ Moreover, Alneyadi et al. reported high levels of stress in especially women healthcare professionals.^38^ It was known that stress was associated with fatigue, fibromyalgia, and burnout for working professionals and breathing exercises may be used for stress coping methods, as well as improving sleep disorders in fibromyalgia.

Several limitations must be acknowledged in this investigation. First, as a self-report study, the results cannot rule out other causes. Second, our sample only represented a small part of FMS patient, therefore generalisations should be made with caution.

The strength of this study is that it is the first study to investigate the effects of CCB exercises to evaluate sleep quality, sexual function, anxiety, and depression scores separately on FMS.

In conclusion, CCB exercises can positively influence quality of sleep, anxiety, depression, and sexual life in patients with FMS. We suggest the incorporation of breathing exercises into the curriculum to balance quality of life programs for women with FMS.

## Data Availability

The dataset produced and examined in this study is not publicly accessible due to privacy and ethical constraints. Kindly reach out to the corresponding author. Our study does not bear any conflict.
